# The active enhancer network operated by liganded RXR supports angiogenic activity in macrophages

**DOI:** 10.1101/gad.242685.114

**Published:** 2014-07-15

**Authors:** Bence Daniel, Gergely Nagy, Nasun Hah, Attila Horvath, Zsolt Czimmerer, Szilard Poliska, Tibor Gyuris, Jiri Keirsse, Conny Gysemans, Jo A. Van Ginderachter, Balint L. Balint, Ronald M. Evans, Endre Barta, Laszlo Nagy

**Affiliations:** 1 Department of Biochemistry and Molecular Biology, University of Debrecen, Debrecen H-4032, Hungary;; 2 The Salk Institute for Biological Studies, San Diego, California 92037, USA;; 3 Myeloid Cell Immunology Laboratory, Vlaams Instituut voor Biotechnologie (VIB), Brussels B-1050, Belgium;; 4 Laboratory of Cellular and Molecular Immunology, Vrije Universiteit Brussel, Brussels B-1050, Belgium;; 5 Laboratory of Clinical and Experimental Endocrinology, Katholieke Universiteit Leuven, Leuven B-3000, Belgium;; 6 MTA-DE Lendület Immunogenomics Research Group, University of Debrecen, Debrecen H-4032, Hungary

**Keywords:** RXR, macrophage, transcription, enhancer, angiogenesis

## Abstract

RXR signaling is predicted to have a major impact in macrophages, but neither the biological consequence nor the genomic basis of its ligand activation is known. Comprehensive genome-wide studies were carried out to map liganded RXR-mediated transcriptional changes, active binding sites, and cistromic interactions in the context of the macrophage genome architecture. The macrophage RXR cistrome has 5200 genomic binding sites, which are not impacted by ligand. Active enhancers are characterized by PU.1 binding, an increase of enhancer RNA, and P300 recruitment. Using these features, 387 liganded RXR-bound enhancers were linked to 226 genes, which predominantly reside in CTCF/cohesin-limited functional domains. These findings were molecularly validated using chromosome conformation capture (3C) and 3C combined with sequencing (3C-seq), and we show that selected long-range enhancers communicate with promoters via stable or RXR-induced loops and that some of the enhancers interact with each other, forming an interchromosomal network. A set of angiogenic genes, including *Vegfa*, has liganded RXR-controlled enhancers and provides the macrophage with a novel inducible program.

## Abstract (precis)

Here, Nagy and colleagues use genome-wide approaches to uncover the activity of RXR, an enigmatic member of the nuclear receptor superfamily. RXR signaling is predicted to have a major impact in macrophages, but neither the biological consequence nor the genomic basis of its ligand activation is known. Integrating RNA-seq, ChIP-seq, GRO-seq, and 3C-seq, the authors unravel the mechanism of RXR-induced transcriptional events in mouse bone marrow-derived macrophages. Importantly, this study uncovers a novel biological activity—angiogenesis—that is promoted by the receptor.

RXR is an unusual and somewhat neglected member of the nuclear receptor superfamily because it is still not known how this receptor interacts with the genome and regulates gene expression upon ligand activation. It is expressed in every cell type and is required for postnatal life in mice (Mangelsdorf et al. 1992; for review, see Szanto et al. 2004). Its presumed main molecular function is to regulate the activity of a dozen or so nuclear receptors. There is also evidence that it can form homodimers and/or have heterodimer-independent signaling capacity (Zhang et al. 1992; Szeles et al. 2010). A key concept regarding RXR signaling is the permissiveness/nonpermissiveness mutually exclusive dual paradigm. According to this, in certain heterodimers such as RXR:PPAR and RXR:LXR, ligand activation of RXR results in transcriptional activation; hence, these are permissive heterodimers, while in other heterodimers such as RAR:RXR, TR:RXR, and VDR:RXR, RXR is suppressed or “subordinated,” and therefore these so-called nonpermissive heterodimers cannot be activated from the RXR side (Germain et al. 2002). Therefore, the activation of all permissive heterodimers present in a particular cell type might lead to pleiotropic gene activation and engagement of potentially conflicting pathways.

The existence of pleiotropy, the role of RXR activation of permissive heterodimers, and the presence and activity of RXR homodimers have been debated and remain largely unresolved.

The fact that certain natural lipids—such as 9-cis retinoic acid (RA), docosahexanoic acid, and phytanic acid—are able to activate RXR gives further support to the biological role of RXR activation in vivo (for a review, see Szanto et al. 2004). There are also potent and selective synthetic compounds such as bexarotone (LG10069) and LG100268 (LG268) (Boehm et al. 1994, 1995) that have been used to dissect the role of the receptor in various biological systems and/or used in therapies. In macrophages, there are several heterodimeric receptors with key cellular roles, such as PPARγ regulating oxLDL uptake and processing, LXR regulating cholesterol efflux and immune function, and NR4A1 (NUR77) regulating inflammatory response. These heterodimeric receptors have been linked to the development of atherosclerosis and also immune function and provide means to reprogram macrophages (for reviews, see Calkin and Tontonoz 2012; Nagy et al. 2012). Therefore, it is important to understand how activation of RXR contributes to these pathways and potentially to novel ones and regulates gene expression in this cell type.

A key issue in understanding any signal-specific transcription factor is the determination of the genomic sites to which it binds and to link those to the target transcripts. Recent advances in genome-wide approaches allow global assessment of histone modifications and transcription factor genomic binding sites (Ostuni et al. 2013). It is done using microarrays or RNA sequencing (RNA-seq) (Mortazavi et al. 2008). However, the temporal changes in steady-state mRNA levels diverge from changes in transcription; hence, simply determining steady-state mRNA levels will not allow easy identification of primary transcriptional events and will not separate those from secondary and tertiary ones (Bhatt et al. 2012). The development of genome-wide localization studies, primarily ChIP-seq (chromatin immunoprecipitation [ChIP] combined with deep sequencing) approaches, aided the determination of transcription factor-binding sites within the context of the chromatin structure on the genomic scale. Such genome-wide studies suggest that enhancers and promoters exhibit distinct chromatin “signatures.” The characteristic signature for enhancers consists of monomethylation of histone H3 Lys4 (H3K4me1), acetylation of histones (H3K9ac and H3K27ac), and binding of the acetyltransferase P300 (Heintzman et al. 2007; Koch et al. 2007; Visel et al. 2009).

In spite of all these developments, the reliable linking of activated enhancers to the regulated gene is still not solved, partly due to the lack of markers linking the genomic binding site to the regulated transcript and partly due to the potentially large distances between the enhancer and the regulated gene. Moreover, the identification of enhancers is even more cumbersome, if not impossible, in the case of signal-dependent transcription factors (such as RXR), which may constitutively occupy genomic sites. In our studies, we tried to solve these issues by combining RNA-seq, ChIP-seq, GRO-seq (global run-on sequencing), and 3C-seq (chromosome conformation capture [3C] combined with sequencing) in a highly integrated way to unravel the mechanism of RXR-induced transcriptional events in mouse bone marrow-derived macrophages (BMDMs) and, as the result of the process, discovered and validated a novel biological activity promoted by the receptor.

## Results

### The transcriptional consequences of RXR activation in murine macrophages

**Figure 1.**
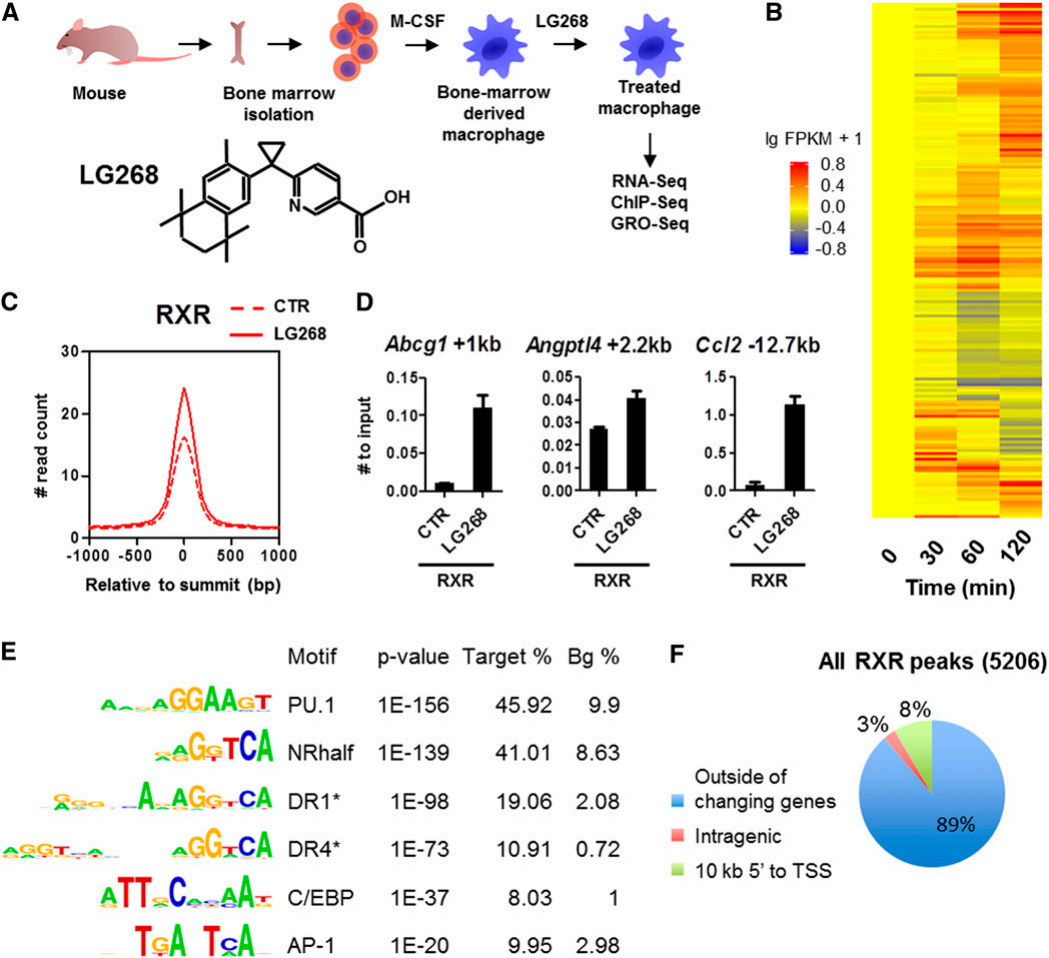
Mapping the transcriptional consequences of selective activation of RXR in murine macrophages. (*A*) The scheme of macrophage differentiation and the chemical structure of LG268. (*B*) Heat map representation of the expression of genes regulated by LG268. Differentiated macrophages were treated with 100 nM LG268 for the indicated time, and total RNA was subjected to RNA-seq gene expression analysis (Supplemental Fig. S1A). Biological replicates had at least 70 million reads each. The presented gene set was filtered at FDR < 0.1 and a more than twofold change. (*C*) Average read distribution of RXR peaks in the presence or absence of LG268. ChIP-seq data were analyzed as shown in Supplemental Figure S1B (peaks were predicted by MACS2, and the consensus of the 2 × 2 replicates was determined by DiffBind); the histogram was generated by HOMER. (#) Normalized throughout the study. (*D*) RXR binding on the indicated individual enhancers confirmed using ChIP-RT-qPCR. Macrophages were treated with 100 nM LG268 for 1 h. The mean and ±SD of triplicate determinations are shown. (*E*) De novo and targeted (asterisk) identification of motifs under RXR peaks from ChIP-seq data using HOMER. “Target %” refers to the ratio of the peaks having the given motif, and “Bg %” refers to the ratio of a random background as described in the Supplemental Material. (*F*) The genome-wide distribution of RXR peaks relative to the TSS of the closest regulated genes identified by RNA-seq (*P* < 0.05; *n* = 823).

We set out to systematically determine the genomic events following RXR ligand activation in BMDMs (Fig. 1A). The synthetic and selective RXR ligand LG268 was applied throughout the studies in a 100 nM concentration. We determined the changing transcripts by RNA-seq (analyzed by the pipeline shown in Supplemental Fig. S1A) and found that selective activation of RXR affects the steady-state mRNA levels of hundreds of genes, as expected. A hierarchical clustering of the top 200 changing genes as a time course is shown in Figure 1B. In order to identify primary RXR targets, we embarked on analyses to determine both the genomic binding events of RXR (RXR cistrome) and the enhancers responsible for the transcriptional changes.

### Determination of the RXR, PU.1, and P300 cistromes and mapping active histone marks in BMDMs, the effect of ligand

By carrying out ChIP-seq experiments, we determined the cistromes of RXR in the absence and presence of its ligand, LG268, and its relationship to the lineage-specific transcription factor PU.1 and the cofactor P300 along with a marker of transcription initiation, H3K4me3, and active histone marks, H3K27ac, H4ac, and H3K4me2, using the algorithm depicted in Supplemental Figure S1B. We determined ∼5200 RXR genomic binding regions. Importantly, this cistrome is not impacted greatly by ligand treatment in 60 min. PU.1 occupied the highest number of peaks (∼30,000), with only a minimal rearrangement upon ligand exposure (Supplemental Fig. S1C). Although the number of RXR-binding regions does not change much upon ligand exposure, peaks gain ∼30% more reads, suggesting that RXR enrichment on the genomic regions is enhanced (Fig. 1C). We confirmed these observations by ChIP and real-time quantitative PCR (RT-qPCR) on binding regions of known directly regulated genes (Fig. 1D; Nagy et al. 1996; Kersten et al. 2000; Repa et al. 2000). Intriguingly, P300 binding follows the genome-wide RXR enrichment, suggesting that P300 is likely to be recruited to the genome upon RXR activation (Supplemental Fig. S1C).

The motif rank order under the detected RXR peaks reported that PU.1 had the most enriched motif, followed by various combinations of repeats, including DR1 and DR4, of the nuclear receptor-binding (half) site. In addition, with somewhat lower abundance, two other macrophage-associated motifs were also detected: C/EBP and AP-1 (Fig. 1E). As far as the genomic distribution of the detected peaks is concerned, ∼90% were found outside of the potentially directly regulated 823 genes identified by RNA-seq experiments (i.e., not within 10 kb of their transcription start sites [TSSs]) (Fig. 1F), suggesting that linking binding sites to regulated genes based simply on proximity will be difficult.

**Figure 2.**
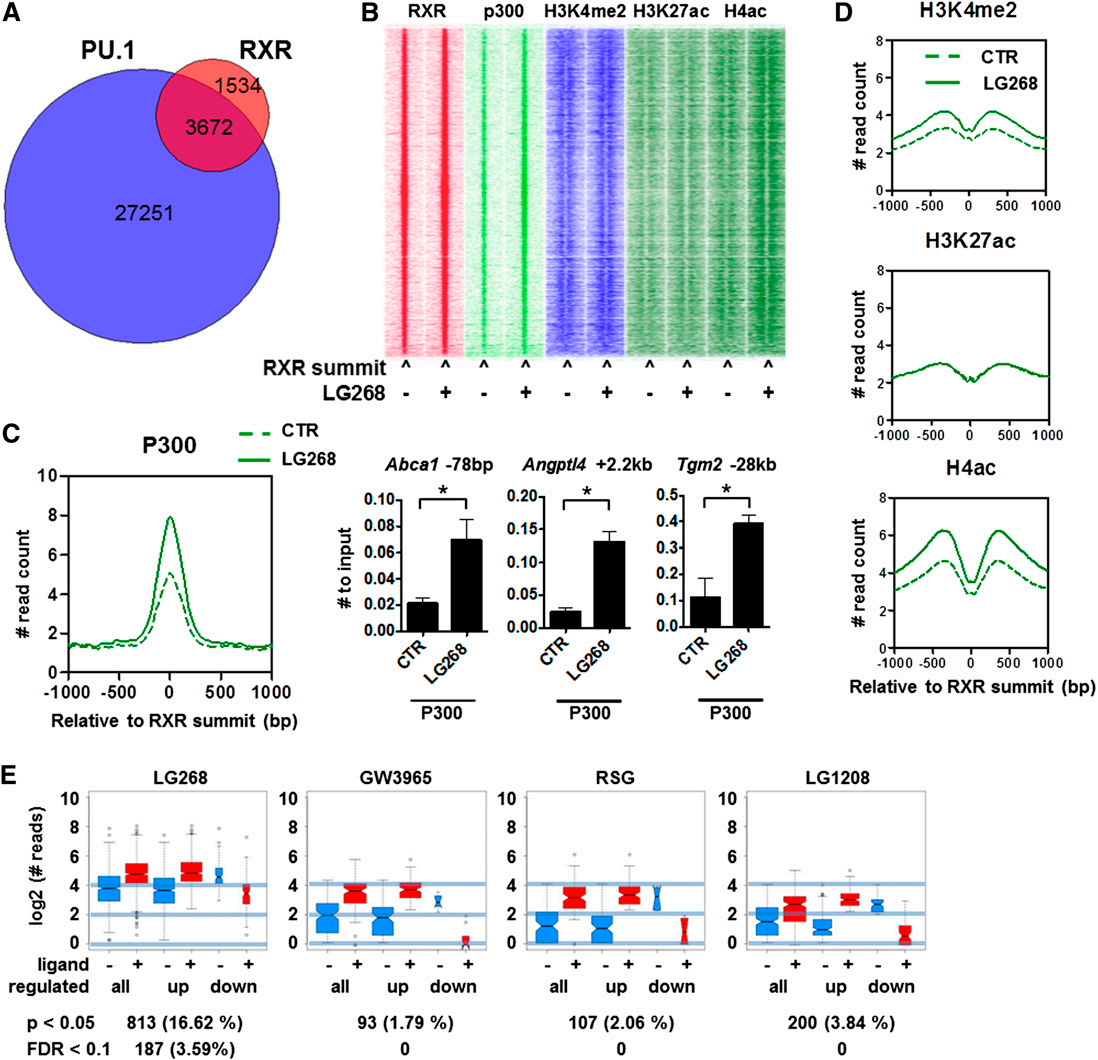
The cistromic interactions of RXR, PU.1, P300, and active histone marks. (*A*) The intersection of cistromes as assessed by number of overlapping peaks is represented as a Venn diagram of RXR and PU.1. (*B*) Heat map representation of RXR, P300, H3K4me2, H3K27ac, and H4ac occupancies in 3-kb windows around the summit of the RXR peaks in the presence or absence of LG268. Read distribution was determined by HOMER, clustering was done by Gene Cluster 3.0 using centered correlation similarity metric with single linkage clustering method, and heat maps were created by Java TreeView in log_2_ scale. (*C*) Read distribution of P300 on RXR peaks in the presence or absence of LG268. P300 binding was confirmed on the indicated individual enhancers using ChIP-RT-qPCR. Macrophages were treated with 100 nM LG268 for 1 h. The mean and ±SD of three biological replicates are shown. Asterisk represents significant difference at *P* < 0.05; *n* = 3. (*D*) Average read distribution of the indicated active histone marks on RXR peaks in the presence or absence of LG268 determined by HOMER. (*E*) RXR enrichments of the significantly changing peaks in the presence of the indicated ligands. Cistrome was determined in the presence or absence of LG268, and the log_2_-normalized read numbers of the significantly changing peaks were plotted by DiffBind. The number of changing peaks and the statistical stringency applied is indicated *below* each plot.

Next, we continued to interrogate the cistromes of PU.1, RXR, and P300 in the ligand-activated state. The RXR peaks overlap with both PU.1 and P300 to a large extent and show increased acetylation (Fig. 2A,B). Global analyses show that ligand activation of RXR leads to P300 recruitment (Fig. 2B,C). These changes could be validated by ChIP-RT-qPCR (Fig. 2C). Importantly, global P300 binding and recruitment could be detected and confirmed at >400 sites using three biological replicates, and no decrease could be observed (Supplemental Fig. S2A). As far as active histone marks are concerned, H4 acetylation and H3K4 dimethylation are increasing, while H3K27 acetylation remains largely unchanged upon ligand activation on the RXR-bound genomic regions (Fig. 2D).

Next, we sought to determine whether some of the potential partner ligands had a detectable effect on the RXR cistrome. Therefore, we determined the RXR cistromes in the presence of a synthetic LXR (GW3965) or PPARγ (RSG) ligand as the activators of the two major suspected heterodimeric partners present in macrophages. We found that neither ligand had a statistically significant effect on RXR’s genome-wide distribution and/or affinity, applying a <0.1 false discovery rate (FDR) threshold (Fig. 2E). Finally, we wanted to evaluate the possibility of whether an endogenous RXR ligand is masking a ligand-induced (re)distribution of the receptor. We reasoned that eliminating the effect of a presumed endogenous ligand by an RXR antagonist (LG1208) would reveal the true unliganded RXR cistrome. However, as shown in Figure 2E, the RXR cistrome does not change upon antagonist treatment either. These findings were confirmed using RT-qPCR (Supplemental Fig. S2B). These data suggest that the RXR cistrome is not changing upon short-term ligand activation and that the RXR selective ligand only increases the receptor’s enrichment at its predetermined binding sites, which typically bind PU.1 and recruit P300. Importantly, the dominant ligands of relevant permissive heterodimers such as PPARγ or LXR do not appear to have an effect on either distribution or affinity.

### Determination of liganded RXR regulated nascent RNA production

The merging of the ChIP-seq and RNA-seq data sets proved to be insufficient to reliably identify the network of primary regulated genes and their enhancers. This is due to the fact that the physical location of the bound transcription factor cannot be linked to the regulated gene. Almost 90% of the RXR peaks are located outside of the 10-kb region upstream of the TSS of the closest regulated gene (Fig. 1F), which is in good correlation with others’ observations (Thurman et al. 2012).

We reasoned that in order to link a liganded RXR-occupied enhancer to the corresponding regulated gene, one needs a high-resolution method, which can provide dynamic temporal information about transcription. The recently recognized fact that nascent RNA production can be detected on both the regulated gene and the enhancer offered a plausible solution (Wang et al. 2011).

**Figure 3.**
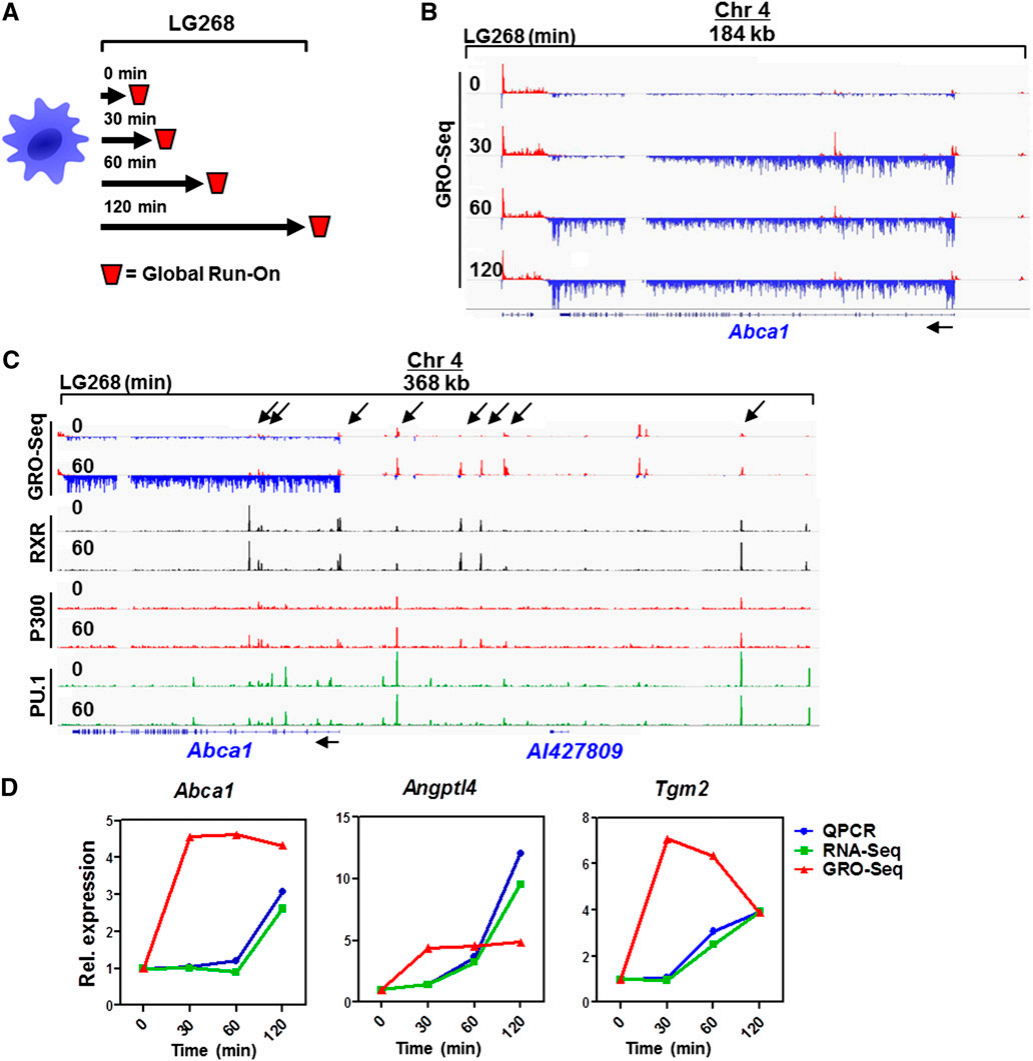
Determination of nascent RNA production upon activation of RXR. (*A*) The scheme of GRO-seq experiments. Cells were treated for the indicated time periods and collected, nuclei were isolated, and run-on sequencing was performed. (*B*) The detected induction of GRO-seq activity on the *Abca1* locus. Nascent RNA produced around the *Abca1* locus is shown. Strand-specific coverage is represented by red and blue. (*C*) Genome browser view of the merge of GRO-seq, and ChIP-seq activity/peaks on the *Abca1* locus. Overlaps of PU.1, RXR, and P300 binding with divergent sites (GRO-seq-positive) are indicated with arrows. (*D*) Comparison of mRNA production dynamics using nascent (GRO-seq) and steady-state (RNA-seq and RT-qPCR) RNA determinations. Macrophages were treated with LG268 for the indicated time period, and specific gene expression was detected with the indicated method. A representative set of experiments is shown. Expression was normalized to the 0 time point.

Therefore, we decided to detect the dynamics of nascent RNA production using GRO-seq. As shown in Figure 3A, we carried out a time-course experiment to determine the sites and dynamics of nascent RNA production upon LG268 activation, a typical example of which is shown for *Abca1* induction in Figure 3B. Importantly, we noticed the presence and induction of nascent RNA transcripts at sites of enhancers, eTranscripts, and also short divergent transcripts (Core et al. 2008). We collectively call these enhancer RNAs (eRNAs). Notably, if one aligns the detected GRO-seq activities with cistromic data, it is easy to recognize that short transcripts overlap with transcription factor-bound regions—in our case, PU.1, RXR, and P300 (Fig. 3C; Supplemental Fig. S3A). A comprehensive analysis of GRO-seq active regions, including gene transcript and eRNA calling, was carried out by a unique algorithm (Supplemental Fig. S3B). We determined that ∼20% of the mouse genome is transcribed (with at least 0.006 RPKM [reads per kilobase per million mapped reads] expression) in this macrophage cell type and identified 10,586 known genes also marked by H3K4me3 and, all together, 25,560 transcripts, which include eRNAs and other noncoding RNAs as well (Supplemental Fig. S3C). Importantly, we found 51,657 GRO-seq divergent sites (>0.2 RPKM expressed) characterized by divergent transcription with a distance <300 bases between the 5′ ends of the transcripts on the two strands. These sites do not necessarily show elongation, just initiation irrespective of transcribed genes and other longer transcripts. One could reasonably assume that this set of genomic regions contains all active promoters and enhancers. Using this approach, we determined changing (induced/repressed) gene transcripts and eRNAs as well. If one plots the changing levels of nascent RNA production of already established direct target genes, the dynamics of the changes are indicative of an immediate induction (Fig. 3D). This also allows easy identification of additional direct targets.

These data further reinforced the notion that the dynamically changing nascent RNA landscape provides clues about direct transcriptional regulation specific for RXR liganding. Furthermore, this allowed us to generate a list of genes most likely being directly regulated by liganded RXR (Supplemental Table S1).

**Figure 4.**
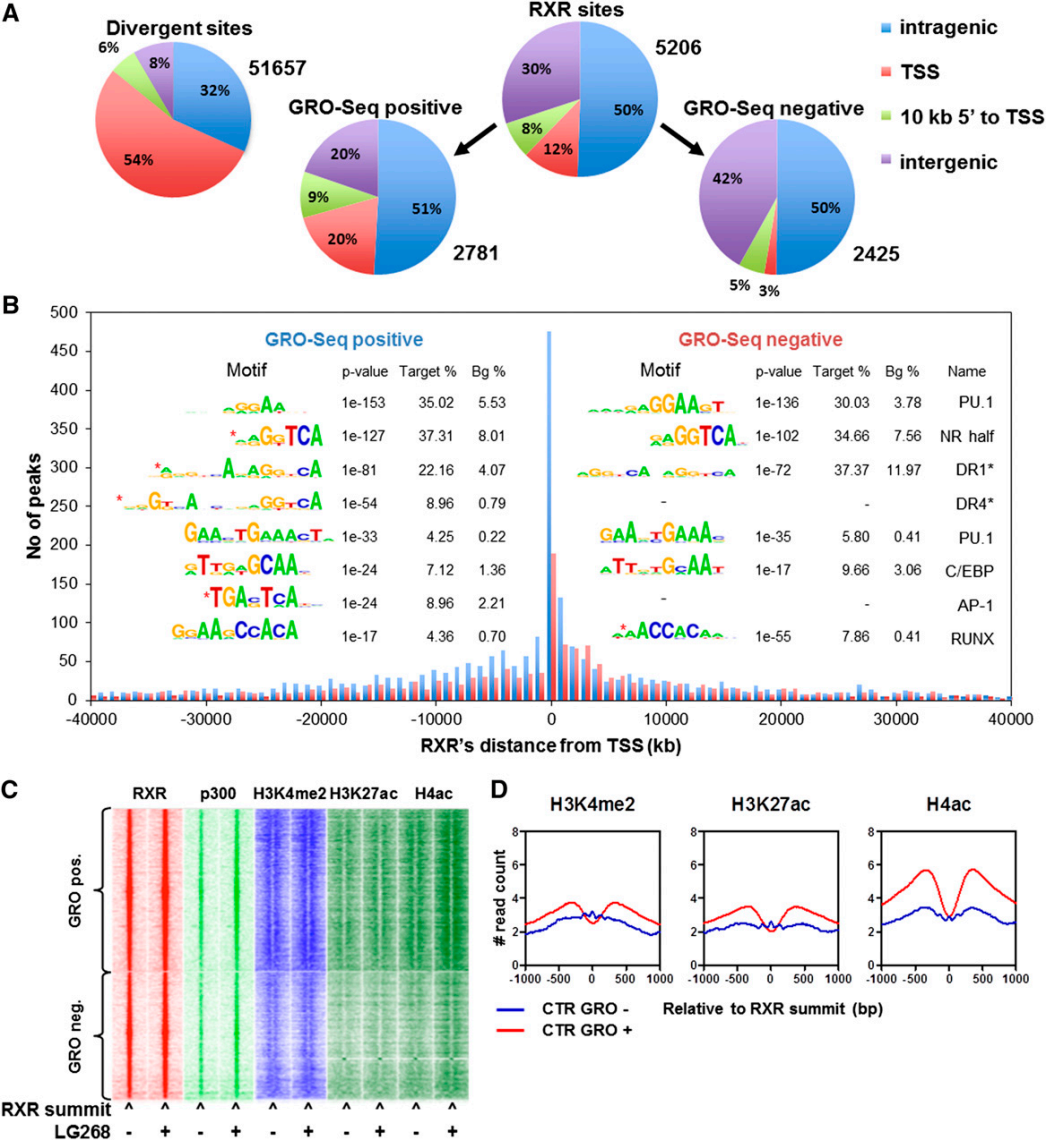
Classification of RXR-binding sites based on GRO-seq activity. (*A*, *top left*) Distribution of the (GRO-seq^+^) divergent sites detected based on genome-wide localization relative to the closest expressed transcripts (>3 kb including 3′ overhang) determined by GRO-seq. Distribution of all RXR sites (*top right*), the RXR sites overlapping with GRO-seq activity (*bottom left*), and the RXR sites not overlapping with GRO-seq activity (*bottom right*) relative to the expressed transcripts. (*B*) Distribution of GRO-seq-positive (blue) and GRO-seq-negative (red) RXR peaks compared with the TSSs of the closest expressed transcripts (defined as in *A*). Columns represent the peak number of the 1-kb distance bins. (*Insert*) De novo and targeted (black asterisk) identification of motifs under GRO-seq-positive (*left* side) and GRO-seq-negative (*right* side) RXR peaks determined using HOMER. Differentially represented motifs are marked by red asterisks. “Target %” refers to the ratio of the peaks having the given motif, and “Bg %” refers to the ratio of a random background as described in the Supplemental Material. (*C*) Heat map representation of RXR, P300, H3K4me2, H3K27ac, and H4ac occupancies in 3-kb windows around the summit of the GRO-seq-positive and GRO-seq-negative RXR peaks in the presence or absence of LG268. Read distribution was determined by HOMER, clustering was done by Gene Cluster 3.0 using centered correlation similarity metric with single linkage clustering method, and heat maps were created by Java TreeView in log_2_ scale. (*D*) Read distribution of the indicated histone marks relative to the GRO-seq-positive and GRO-seq-negative RXR cistromes determined by HOMER.

As far as the active enhancers are concerned, we classified the identified ∼5200 RXR-binding sites into two categories using an algorithm (pipeline depicted in Supplemental Fig. S4A): (1) enhancers that overlap with a GRO-seq-positive region (divergent site; 2781) and (2) enhancers that do not overlap with GRO-seq activity (2425), as shown in Figure 4, A and B). This classification showed an enrichment of RXR peaks within the proximity of the closest TSS (<10 kb from the TSS), suggesting that our assumption was likely correct and that we were identifying the functional/active binding regions and efficiently separating those from the silent/nonactive/parking ones. The distribution of GRO-seq-positive and GRO-seq-negative RXR-bound regions relative to the nearest TSS shows a bias toward upstream regions in cases of the positive ones. Most interestingly, the enriched motif distribution under these peaks also showed characteristic differences. The GRO-seq-positive RXR sites showed an enrichment for DR1 and DR4 (binding sites of RXR:PPAR and RXR:LXR heterodimers, respectively) as well as AP-1 sites when compared with the GRO-seq-negative sites (Fig. 4B).

GRO-seq-positive RXR-binding sites are characterized by an increased H3K27ac, H4ac, and also H3K4me2 histone marks when compared with negative ones (Fig. 4C,D). These provided further support to the notion that these sites are indeed functionally distinct and likely represent active enhancers.

**Figure 5.**
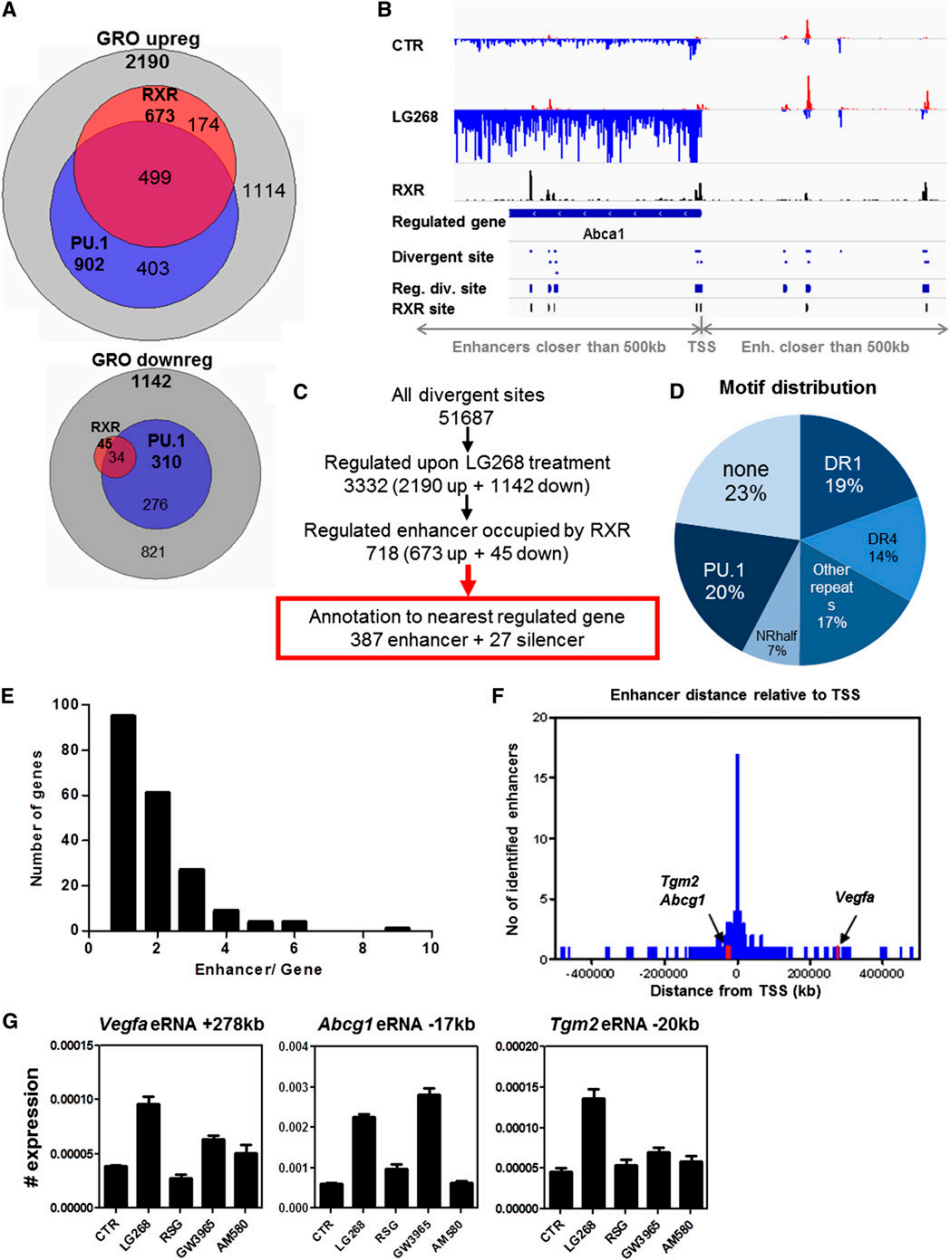
Identification of RXR-regulated enhancers and linking those to regulated genes. (*A*) Overlap of sites of changing GRO-seq activity and cistromes of liganded RXR and PU.1: increasing GRO-seq activity (*top*) and decreasing GRO-seq activity (*bottom*). (*B*) Scheme of annotation criteria on the *Abca1* locus. This includes increased (or decreased) nascent transcript production of a gene and increased (or decreased) divergent transcription of a region occupied by RXR. (*C*) Flow of annotation of GRO-seq-positive RXR-binding regions with changing enhancer transcription to the closest gene with changing expression. See also Supplemental Figure S4A. (*D*) Motif distribution of the annotated 387 RXR-bound enhancers determined by HOMER and fuzznuc as described in the Supplemental Material. Note that additional PU.1 sites can be found in conjunction with other motifs, which are not included here. (*E*) Distribution of the number of enhancers assigned to the closest gene; the *Y*-axis shows the number of genes having the number of enhancers (*X*-axis). (*F*) Distribution of enhancers relative to TSSs of the identified directly regulated genes. (*G*) RT-qPCR analysis of *Vegfa*, *Abcg1*, and *Tgm2* eRNAs. Differentiated macrophages were treated with LG268, RSG, GW3965, and AM580 for 1 h, and eRNAs were determined. Data represent mean and ±SD, with expression normalized to *Rplp0*.

A closer look at the up-regulated GRO-seq-positive regions revealed that those that overlap with RXR peaks overlap with a significant fraction of PU.1 regions as well (Fig. 5A, top). The regions of down-regulated GRO-seq activity have much fewer RXR peaks but show overlap with PU.1 regions to a larger degree as well (Fig. 5A, bottom). These latter analyses showed that there are very few liganded RXR-occupied negative binding sites or silencers, suggesting also that liganded RXR is predominantly a transcriptional activator. Next, we matched up the divergent GRO-seq sites with RXR sites (Fig. 5B; Supplemental Fig. S5A). The identified 51,657 divergent GRO-seq sites likely contain all TSSs, short divergent transcripts, and eRNAs. More than 3300 of these change upon RXR ligand activation, and 718 overlap with RXR-binding sites as well (Fig. 5A,C). We identified 252 regulated genes to which we could assign 414 RXR-bound regions, 387 enhancers, and 27 silencers using the criteria set by us (Fig. 5B,C; Supplemental Fig. S4A).

The distribution of the various motifs within these potentially active enhancers show that ∼50% can be classified as a nuclear receptor-binding site repeat, another 20% is PU.1 only, and a quarter is other or unknown (Fig. 5D). The number of enhancers per gene ranges from one to nine (Fig. 5E). The distribution of these putative enhancers relative to the TSS is fairly symmetrical, and they span long distances, with only 16.54% found within 10 kb upstream of the TSS (Fig. 5F). The identified 387 enhancers have a pronounced increase in RXR occupancy, P300 recruitment, and H4ac, while H3K4me2 and H3K27ac are not different upon ligand treatment (Supplemental Fig. S4B).

Taking these together, we (1) uncovered the core active enhancer network operated by liganded RXR; (2) separated these sites from nonfunctional, silent/parking RXR-binding sites; and (3) paired the active enhancer network with the regulated genes using a set of criteria.

### Functional validation of novel distant and long-range enhancers

Before we embarked on molecular validation of the enhancers, we intended to assess the contribution of the activated partner receptors to the induction of various RXR-regulated genes and eRNAs also. Therefore, we determined the induction of steady-state RNA levels of selected genes. We found that *Vegfa* and *Hbegf* were induced primarily by RXR liganding with some activity by RAR, *Tgm2* and *Ccl6* were both induced by RXR and RAR liganding, *Abcg1* was induced by RXR and LXR, and *Angptl4* was induced by the RXR and PPARγ ligands (Supplemental Fig. S5).

Interestingly, primarily RXR-induced eRNA production could be detected on an enhancer assigned to *Vegfa* or *Tgm2*, while an enhancer of *Abcg1* also showed robust LXR ligand activation, as expected (Fig. 5G). These data suggested that the eRNAs can be easily validated and show ligand induction similar to the regulated genes and therefore most likely are linked.

For functional validation, we chose 45 newly identified regulatory regions assigned to 23 genes (Supplemental Table S1). We selected 30 distant enhancers (>10 kb from the TSS), which earlier studies performing traditional enhancer analyses (Schwartz et al. 2000) would likely have missed, including one of ours (Nagy et al. 1996). In addition, we sought to validate 15 more proximal enhancers (<10 kb from the TSS).

Next, we cloned the 45 putative *cis*-regulatory elements (35 enhancers and 10 silencers) in front of a luciferase reporter gene and measured their transcriptional activity in COS1 cells in the presence of combinations of receptors and ligands. We clustered the enhancers based on the induction patterns obtained in the transient transfection-based reporter system, which is detailed in the Supplemental Material (Supplemental Fig. S6). These analyses show that the identified and tested enhancers are RXR-specific enhancers but also complex and very versatile, allowing combinatorial and context-dependent regulation of the genes by distinct dimers. The context is determined by the receptor expression profile and probably also lineage-specific transcription factors and cofactors. In addition, a single gene is likely to have distinct types of enhancers.

### The genome architectural context of RXR-regulated gene expression

Once we determined the RXR-regulated genes and linked at least a subset of them to their enhancers (some of them are long-range ones), we wanted to get some insight into whether these regulatory units are confined to known features of genome architecture.

Therefore, we decided to explore the genome architectural context of RXR signaling, asking the question of whether RXR signaling is confined by known structural features of genome organization and/or contributes to reorganization of these. We determined the cistromes of CTCF and a member of the cohesin complex (RAD21) in the absence or presence of ligand-activated RXR. We found ∼30,000 peaks for CTCF and ∼24,500 for RAD21 (Supplemental Fig. S1C); the latter showed some increase upon ligand activation. Next, we determined an overlap of ∼12,660 peaks representing similarly high CTCF and RAD21 occupancy (with peaks having a MACS2 score >15 and having less than threefold difference). We considered these as boundaries of functional domains, as suggested by others (Merkenschlager and Odom 2013; Sofueva et al. 2013). We identified 10,204 such functional domains by pairing the CTCF and RAD21 copeaks with the closest ones with similar scores (Supplemental Material).

**Figure 6.**
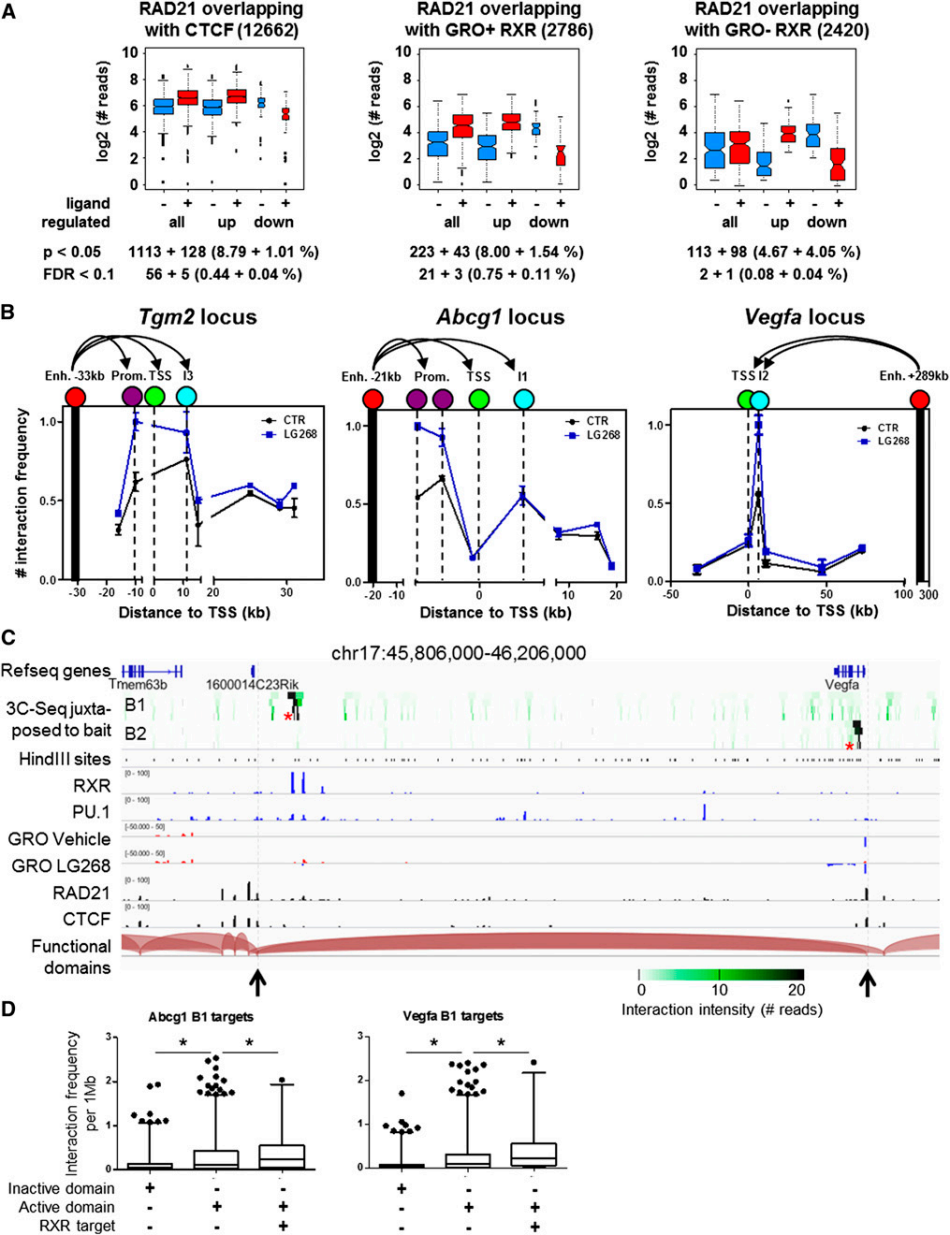
Functional characterization of short- and long-range liganded RXR-regulated enhancers. (*A*) Changing RAD21 peaks in the presence of 100 nM LG268 on CTCF-binding sites (*left*) and GRO-seq-positive (*middle*) and GRO-seq-negative (*right*) RXR-binding sites. The log_2_-normalized read numbers of the significantly changing peaks are plotted. The number of changing peaks and the statistical stringency applied are indicated *below* each plot. (*B*) 3C-RT-qPCR measurements in the presence or absence of LG268 on the *Tgm2*, *Abcg1*, and *Vegfa* loci. Constant primers were designed to the enhancers and are represented by black columns (anchor points). Red circles represent the enhancers, purple ones are the promoter regions, TSSs are depicted with green, and intronic regions are highlighted by cyan circles. The mean and ±SD of triplicate determinations are shown. Representative examples are shown for each locus. (*C*) Genome browser view of the *Vegfa* locus containing the proximal interacting regions of the intergenic (B1) and intronic (B2) baits and the loop predictions generated based on CTCF/RAD21-cobound regions. 3C-seq heat map (green scale) was made as described in the Supplemental Material. Asterisks show the sites of the specific baits. GRO-seq and ChIP-seq for the indicated factors are shown. Black arrowheads and gray dashed lines indicate the predicted domain borders. (*D*) Box plot representation of the distribution of interaction frequency of *Abcg1* B1 enhancer (chr17: 31,172,998–31,173,656) and *Vegfa* B1 enhancer (chr17: 45,890,060–45,890,829) determined by 3C-seq. The interchromosomal interactions of these enhancers were determined as described in the Supplemental Material. The detected interactions were mapped onto 1-Mb fragments covering the mouse genome. Interaction frequency was determined by expressing the read number per 1 Mb normalized to 1000 reads. Genome regions (inactive, active, and RXR target) were qualified as described in the Supplemental Material. Unpaired two-tailed *t*-test analysis was used to determine significant differences. Asterisk represents significant difference at *P* < 0.0001.

The median length of such domains was 81.15 kb, while the average was 149.98 kb. The neighboring domains then were merged into active domains with <100-kb distances between them. We could designate almost 700 such domains, which have a median length of 1.1 Mb and an average length of 1.88 Mb. If one overlays this architectural domain structure with active RXR-binding sites and regulated genes that we identified (Fig. 5C), one can find that 203 out of the 252 (80%) identified RXR-regulated genes along with their enhancers fall on such functional domains. In addition, one can identify ∼1113 CTCF genomic binding regions on which RAD21 (cohesin) binding is enhanced and 128 regions on which RAD21 binding is reduced upon RXR ligand treatment (Fig. 6A, left). Moreover, RAD21 (cohesin) binding is more enhanced on active RXR enhancers (GRO-seq^+^ ones) when compared with nonactive ones (GRO-seq^−^ ones) (Fig. 6A, middle and right). Importantly, 40% (84 out of 203) of regulated genes have induced RAD21 on their enhancer and/or CTCF-binding sites. Collectively, these data suggest that most if not all RXR enhancers act inside functional domains and that activation of the receptor contributes to formation of such domains by stabilizing the genomic architecture and, in some cases, even inducing the binding of enhancers to promoters. Next, we validated molecularly the interactions in such functional domains and the impact of ligand on these for long-range enhancers of *Abcg1*, *Vegfa*, and *Tgm2* using RT-qPCR-based 3C (Fig. 6B). These data clearly documented that the enhancers identified by the combination of RNA-seq, ChIP-seq, and GRO-seq act in functional domains, loop to the promoter, and can be readily functionally validated using transient transfection and 3C as well.

Finally, we asked the question of whether any of these enhancers communicate with other *cis*-regulatory elements or functional domains in the genome. Thus, we carried out 3C-seq using pairs of baits located in or close to these regions. We could detect proximal and also long-range interactions (for details of the analyses, see the Supplemental Material). At the *Vegfa* locus, we could detect interactions between the distant enhancer and the neighboring enhancers as well as the intronic region with remarkable specificity (Fig. 6C). Similar results were obtained in the case of the *Tgm2* and *Abcg1* loci (Supplemental Fig. S7A,B). Importantly, we also detected interchromosomal interactions with much less frequency (at least 50-fold less) though. In order to provide statistical context to these findings, we compared the interaction frequency of a given bait with inactive topological domains, active domains devoid of RXR-regulated regions, and active domains with RXR-regulated regions. As shown in Figure 6D in the case of *Abcg1* and *Vegfa*, there is significant difference between the frequencies of such interactions. Similar results were obtained with additional enhancer of these and the *Tgm2* gene. This suggests that active RXR enhancers interact with other active genomic regions and with an even higher likelihood with other RXR-regulated ones. These formally suggest that the active RXR enhancers form an interchromosomal hub or network.

### Determination of the impact of RXR activation on the angiogenic capacity of macrophages

Finally, we wanted to see whether some of the identified novel transcriptional pathways could be validated biologically. The functional annotation of the genes controlled by liganded RXR enhancers assigned a large number of genes into the angiogenesis category (Supplemental Fig. S4C). These include *Vegfa*, *Hbegf*, *Litaf*, and *Hipk2*. Therefore, we decided to functionally test this activity.

**Figure 7.**
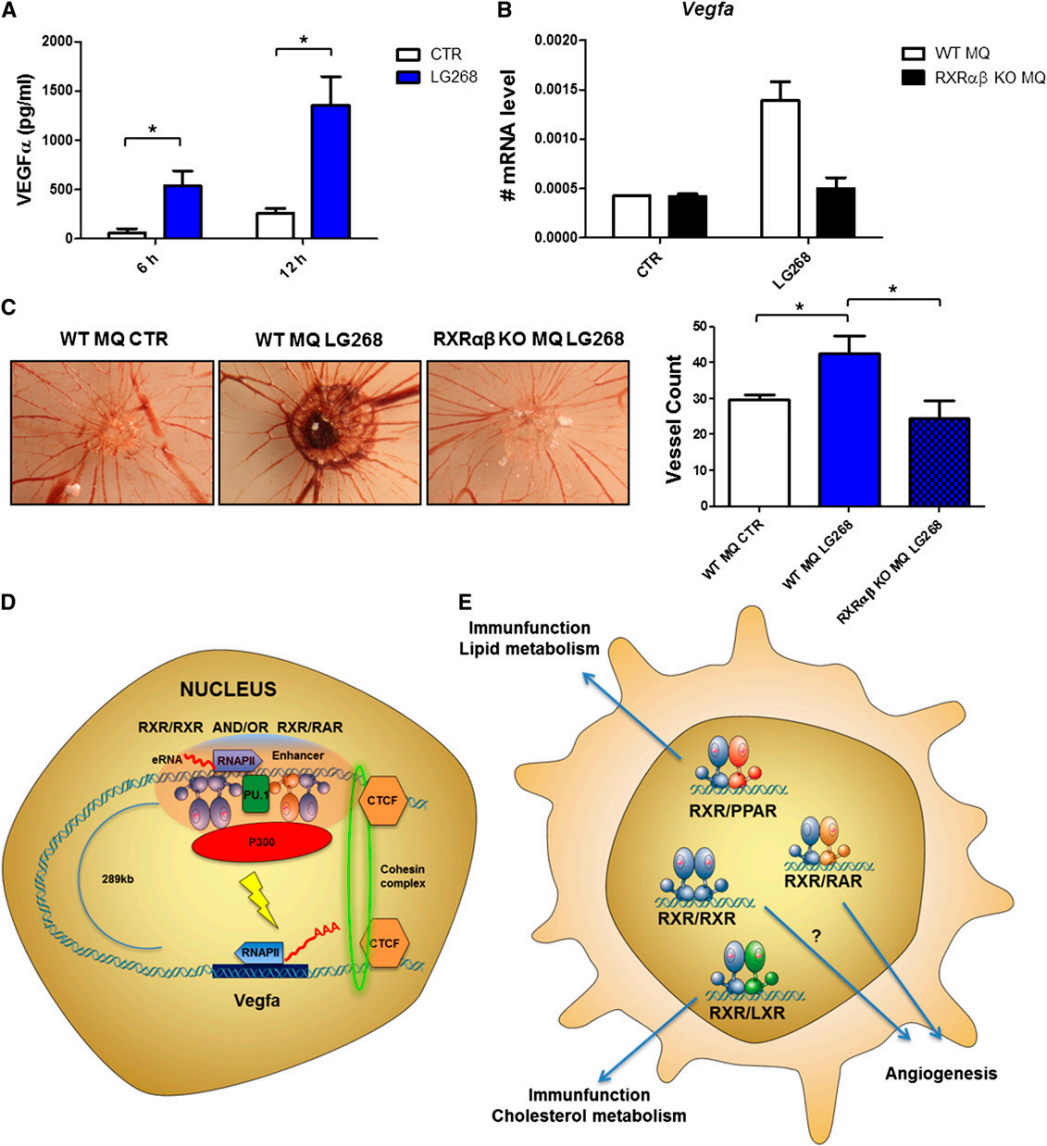
RXR activation leads to increased angiogenic activity in macrophages. (*A*) VEGFα protein levels determined from supernatants of macrophages treated with LG268 for the indicated periods of time determined by ELISA. The mean and ±SD of triplicate determinations are shown. Asterisk represents significant difference at *P* < 0.05; *n* = 3. (*B*) *Vegfa* mRNA levels determined using RT-qPCR in wild-type (WT) and RXRα/β knockout (KO) macrophages. The mean and ±SD of duplicate determinations are shown. (*C*) Macrophage angiogenesis activity was determined using CAM assay in the presence or absence of LG268 using wild-type and RXRα/β knockout macrophages. Representative images (*left*) and bar chart of quantification (*right*) are shown. The mean and ±SD of three biological replicates are shown. Asterisk represents significant difference at *P* < 0.05; *n* = 3. (*D*) The molecular mechanism through which *Vegfa* is regulated in macrophages. *Vegfa* harbors a set of very-long-range enhancers. The enhancers are marked by the lineage-determining transcription factor PU.1 and are able to recruit RXR presumably as a heterodimer with RAR or in a homodimeric form. Upon ligand stimulation, P300 is recruited, and eRNA production is highly increased. These elements can get into the close proximity of the gene promoter by looping, which is enhanced in the presence of the RXR activator (LG268) and leads to increased gene expression. This regulatory unit is bordered by CTCF/cohesin (RAD21) cobinding, which is thought to contribute to the topological domain structure of mammalian genomes and stabilizes chromatin loops. (*E*) Schematic representation of the various nuclear receptor dimers and their biological significance in macrophages. PPAR/RXR are known to inhibit the inflammatory response upon activation by either side as a permissive heterodimer, while the LXR/RXR heterodimer regulates inflammatory response, cholesterol metabolism, and triglyceride biosynthesis. Our results shed light on the effect of putative, permissive, RAR/RXR, or possibly RXR/RXR dimers in the angiogenesis program of macrophages, most probably through the regulation of *Vegfa* and/or *Hbegf*.

First, we determined the excreted VEGFα protein levels from cell supernatants (Fig. 7A) using different ligands and could show that RXR induced this protein, as was suggested by our gene expression measurements as well (Supplemental Fig. S5). Using RT-qPCRs, we could show the RXR requirement using RXRα/β double-knockout macrophages (Fig. 7B). To test whether activation of RXR promoted angiogenesis in an in vivo relevant setting, we performed a chorioallantoic membrane (CAM) assay using macrophages pretreated with RXR agonist. These cells showed significantly increased angiogenic activity, which was not detectable in RXRα/β double-knockout macrophages (Fig. 7C). These data suggest that RXR activation can program macrophages toward a distinct cell-autonomous angiogenic phenotype (Fig. 7D,E).

## Discussion

The ultimate goal of studying the function of a particular transcription factor is to discern its primary biological action in the entire genome comprehensively. In principle, this should be done by defining the genomic binding regions and determining the genes proximally regulated in a given cell type. Using computational tools, one can identify potential RXR-binding sites on the scale of several times 100,000 in the mouse genome. When all of the ChIP-seq data available from fat, from liver, and now in macrophages are combined, the combined number of genomic regions bound by RXR is a few times 10,000. Still, in macrophages, the number of genomic regions is ∼5200, but the number of regulated genes by liganding the receptor is a few times 100. This is further complicated by the fact that primary targets are hard to reliably identify by simply measuring steady-state mRNA levels. So the question remains: How can one find the regulated genes and the linked receptor-bound enhancers? We attempted to do this by integrating genome-wide analyses.

### Integration of genome-wide localization and nascent RNA production data identifies active enhancers

A key premise of the work presented here is that genome-wide localization studies, predominantly ChIP-seq experiments, can be intersected with data from nascent RNA determinations such as GRO-seq time courses, which then enables one to comprehensively annotate cistromes and identify active enhancers (Hah et al. 2013; Lam et al. 2013). This relies on the assumption that in vitro (nuclear run-on) determination of the activity of RNA polymerases (RNAPs) is a reliable indicator of transcriptional activation. This has been proven in other systems and leads to the identification of active enhancers in other cell types (Koch and Andrau 2011; Wang et al. 2011; Bonn et al. 2012), although systematic comparisons or cross-validation with other methods such as subcellular RNA fractionation (Bhatt et al. 2012) has not been done. The usage of GRO-seq for the determination of direct transcriptional responses has two major advantages: (1) The dynamics of the nascent RNA levels depends only on the rate of RNAP activity, and therefore it is matching the expected time course of a directly regulated gene. Our data are clearly demonstrating this because known and established direct target genes (*Abca1*, *Abcg1*, *Angptl4*, and *Tgm2*) as well as newly identified ones (i.e., *Vegfa* and *Hbegf*) show an immediate induction when assessed by GRO-seq rather than a complex, often delayed one determined by RNA-seq or RT-qPCR (Fig. 3D). (2) Enhancers contain engaged RNAPs, and their activity can be revealed by GRO-seq in the form of a typically few-hundred-base-pair-long divergent transcript, termed eRNA. The existence of such transcripts has been shown before (Kim et al. 2010; Wang et al. 2011). Although the mechanism of their production and role in transcription is far from being clarified, it can be reasonably assumed that they represent active enhancers, as the presence of an engaged RNAP strongly indicates it. Furthermore, if the changes of nascent RNA production (increase or decrease) at these sites show correlation with the stimulating signal, it is likely to be associated with or the consequence of the activity of the signal-specific transcription factor. A key limitation of today’s technology is the lack of high-throughput validation of the identified enhancers. Nonetheless, our high rate of success in validating such enhancers with low-throughput enhancer trap approaches, 3C-seq and 3C-RT-qPCR, suggests that signal-specific changes in eRNA production can be used to filter active enhancers from the general pool of genomic binding regions. Altogether, this also means that this combined approach can serve as proof of concept and a model to tackle similar problems with other signal-specific transcription factors. A remarkable feature of the approach is that it allowed the identification of active enhancers in spite of the fact that these represent a fraction of the binding regions (not more than 10%–15% by using our stringent criteria), including very-long-range enhancers. It is also interesting that the enhancers’ distribution relative to the TSS appears to be rather symmetrical instead of being much biased to the upstream regions, suggesting that one needs to look in both directions and also far away to identify the true enhancer controlling a particular gene in a given cellular context. A cautionary conclusion of this study is that it is very likely that many of the enhancers identified with less comprehensive methods might need to be revisited.

### RXR colocalizes with PU.1 and recruits P300 as a cofactor to its binding sites

Our data showed that RXR has >5200 binding regions in macrophages. These peaks are likely to be RXRα-bound because this is the dominant subtype present in macrophages. However the used antibody is pan-RXR-specific and would recognize all three subtypes. Most of these sites contain nuclear receptor half sites (AGGTCA), suggesting that the localization of RXR uses nuclear receptor-mediated direct DNA binding. In addition, two independent lines of evidence suggest that there is an intimate relationship between the lineage-determining factor PU.1 and RXR: (1) 45% of the RXR peaks contain PU.1 sites, and (2) there is a significant overlap between RXR and PU.1 cistromes, as more than two out of three of RXR-binding regions overlap with PU.1 peaks. This is in agreement with our anticipation based on previous reports by Lazar and colleagues (Lefterova et al. 2010) and Liu and colleagues (Pott et al. 2012), who suggested that binding regions for PPARγ, a heterodimerization partner of RXR, colocalizes with PU.1. This finding supports the pioneering or bookmarking factor concept put forward by several investigators (for a review see, Zentner and Scacheri 2012) to explain various interactions and genome-wide colocalization between lineage-specific and signal-specific transcription factors, suggesting that the lineage-specific factor opens up or marks particular regions in the genome, which then allows or even facilitates the binding of the signal-specific factors. Although the sequence of events or the mechanisms of such interactions are unknown, our data are fully compatible with such a scenario. An additional line of evidence supporting this is that there is only a 14% overlap between our RXR-binding regions and the ones found in 3T3L1 cells differentiating into adipocytes (Nielsen et al. 2008). Our work further extended these studies and suggests that many if not all RXR heterodimers at least in part colocalize with PU.1 in macrophages and that upon ligand treatment, PU.1 is at least partially released and therefore might not be required for further binding or transcriptional activity.

Another unexpected aspect of the cistromic interactions identified is that P300 binding is dynamically recruited upon the RXR ligand treatment to most of the active RXR sites. Two conclusions can be drawn from these findings: (1) P300 is major cofactor of mediating transcriptional activity by RXR in BMDMs in the steady state. (2) Liganded RXR-regulated transcription complexes use P300 as one and potentially the major cofactor, and thus P300 is likely to be responsible for the acetylation of histone H4K5/8. This is consistent with the initially proposed role for this protein in nuclear receptor signaling (Chakravarti et al. 1996) and with the more recent findings (Jin et al. 2011) but also suggests that P300 is likely to be specific for this signaling pathway in this cell type.

A key issue with signal-specific transcription factors is whether the signal contributes to the (re)distribution of the particular transcription factor. In case of the RXR-containing receptor dimers, the accepted view is that RXRs bind to the genome in both the absence and presence of ligand (Boergesen et al. 2012). Our findings further support this view now by adding a genome-wide perspective and show that the only effect RXR ligand has on the genome-wide distribution of the receptor is that the enrichment on the preformed binding sites increases. These data obviously have to be interpreted within the context of ChIP experiments, meaning that the sum of all binding in all cells is detected in the time frame of the 40-min cross-linking used. This method does not allow the construction of a more dynamic and/or higher-resolution picture of the receptors’ activation and mobility. Methods using shorter time resolution show a much more dynamic behavior of the receptor though (Brazda et al. 2014). Therefore, our interpretation of the data is that RXRs’ genome-wide localization is determined by its own DNA-binding capacity, which allows it to find preformed sites in the genome to which it can bind, and this is facilitated by additional factors such as a lineage-specific factor; i.e., PU.1. The increased affinity that is observed is either the reflection of more cells being involved in the response or a higher affinity for the binding site. However, the experimental approaches used are not able to determine the relative contribution of these two mechanisms. We also excluded the possibility that an endogenous ligand plays a major role in directing RXRs to its genomic binding sites by using an antagonist. This behavior is in stark contrast to that of steroid hormone receptors such as GR or estrogen receptor (ER), whose genomic binding is dictated by the addition of ligand (Carroll et al. 2005; Welboren et al. 2009). As far as the genome architectural context of the RXR-regulated enhancers and the regulated genes are concerned, we could show that the vast majority of enhancers and their regulated genes are confined to functional domains bordered by CTCF/cohesin (RAD21) complexes, suggesting that their activity localized to these loops (Supplemental Fig. S8). In addition, the rearrangement of such loops is moderately though but impacted by ligand activation. Curiously though, our 3C-seq analyses also revealed that the functional domains and the enhancers within them interact with each other even on different chromosomes. The functional significance of such interactions is not clear but might suggest the existence of RXR- or partner-specific transcription foci or factories inside the nucleus.

### Characteristics of the liganded RXR-operated enhancer network

Our data show, as expected, that the two major permissive heterodimers in BMDMs are RXR:LXR and, to a lesser extent, RXR:PPAR, as shown by the ready induction of their established target genes, *Angptl4*, *Abca1*, and *Abcg1*, and the motifs identified within their RXR peaks being RXR:PPAR-specific (DR1) and RXR:LXR-specific ones (DR4). However, our analyses also uncovered a set of regulated genes, including *Tgm2*, and novel ones, such as *Vegfa* and *Hbegf*, which could not be induced efficiently by ligands for permissive heterodimers, only the RXR-selective LG268 or ligands activating the RAR receptor. This raises the possibility of the existence of permissive RXR:RAR heterodimers or a complex regulatory mechanism/enhancer allowing activation by either RAR or RXR-activating ligands (Fig. 7D).

Underpinning this complexity, the enhancers identified for the regulated genes show a large degree of functional versatility and can be grouped into four broad categories. Cluster A requires the presence of liganded RXR; another (cluster B) is best activated if both RXR and one of its partners is expressed; a third (cluster C) is mediating RXR-specific signaling, provided RXR is expressed at a significantly higher level than its partners; and the enhancers in the fourth (cluster D) work with multiple combinations of dimers (Supplemental Fig. S4). A further level of complexity is that a given gene (i.e., *Vegfa* or *Abca1*) has enhancers from more than one category.

All of these support that RXR has a unique genomic effect that cannot be recapitulated by any ligand or combination of ligands. This is mechanistically served by activation of permissive heterodimers such as LXR:RXR and PPAR:RXR and activation of enhancers inducible by RAR as well. In addition, some of the eTranscript inductions appear to be specific for RXR.

This and the facts that, using our most stringent criteria, we identified only 226 induced genes, and only 7.4% of the identified binding sites are active enhancers suggest that activation of RXR is tightly controlled in this cell type and leads to distinct and selective and not pleiotropic gene expression. Unexpectedly, a network of enhancers that could be linked to a set of genes—including *Vegfa*, *Hbegf*, *Hipk2*, *Litaf*, *Cxcl2*, and *Foxo3*, which have been clearly linked to angiogenesis—was also revealed.

The regulation of *Vegfa* by RXR is intriguing because it appears to use a set of very-long-range enhancers (270 kb downstream) and shows RXR/RAR specificity, including RXR ligand-induced looping (Fig. 7D). We presented here a novel integrated approach to identify functional enhancers and link those to primarily regulated genes for a signal-dependent transcriptional factor, liganded RXR. This integrated approach revealed that ligand stimulation of RXR activates only a small fraction of the DNA-bound molecules confined by CTCF/cohesin-delimited functional domains (Supplemental Fig. S8) and leads to a distinct gene expression program, which results in increased angiogenic potential and might be a valid macrophage reprograming/therapeutic target (Fig. 7E).

## Materials and methods

### Materials

The following ligands were used: LG268 and LG1208 were gifts from M. Leibowitz (Ligand Pharmaceuticals), RSG and AM580 were from Sigma, and GW3965 was a gift from T.M. Wilson (GlaxoSmithKline).

### Differentiation of BMDMs

Isolation and differentiation were completed as described earlier (Barish et al. 2005).

### RNA-seq

The RNA-seq library was prepared from two biological replicates by using a TruSeq RNA sample preparation kit (Illumina) according to the manufacturer’s protocol. Analysis was carried out as described in the Supplemental Material.

### ChIP

ChIP was performed as previously described (Barish et al. 2010), with minor modifications. Libraries were prepared by Ovation Ultralow Library Systems (Nugen) from two biological replicates according to the manufacturer’s instructions. ChIP-seq analysis and domain prediction were carried out as described in the Supplemental Material.

### GRO-seq

GRO-seq and library preparation were performed as described earlier (Core et al. 2008; Hah et al. 2011), with limited modifications. Libraries were generated from two biological replicates of BMDMs treated with 100 nM LG268. Analysis was carried out as described in the Supplemental Material.

### RT-qPCR

RNA was isolated with Trizol reagent (Molecular Research Center). RNA was reverse-transcribed with Tetro reverse transcriptase (Bioline). Transcript quantification was performed by RT-qPCR reaction using SYBR Green dye (Diagenode). Transcript levels were normalized to Ppia or Rplp0.

### 3C

3C experiments were completed as described previously with minor modifications (Miele et al. 2006). The detailed protocol is available in the Supplemental Material.

### 3C-seq

Experiments were carried out as previously described (Stadhouders et al. 2013). For details, see the Supplemental Material.

### Transient transfection

Enhancer sequences were PCR-amplified from a BAC or genomic DNA and cloned into pUC18 HSV TK-LUC. Transient transfections were carried out as previously described (Szanto et al. 2010).

### ELISA

ELISA experiments were carried out according to the manufacturer’s instructions (R&D Systems).

### CAM assay

CAM assays were performed as described previously (Movahedi et al. 2008). The detailed protocol is presented in the Supplemental Material.

### Data access

Sequencing data were submitted to Sequence Read Archive under accession number SRP019970.

## References

[B1] Barish GD, Downes M, Alaynick WA, Yu RT, Ocampo CB, Bookout AL, Mangelsdorf DJ, Evans RM 2005 A nuclear receptor atlas: macrophage activation. Methods Enzymol 19: 2466–2477 10.1210/me.2004-0529 16051664

[B2] Barish GD, Yu RT, Karunasiri M, Ocampo CB, Dixon J, Benner C, Dent AL, Tangirala RK, Evans RM 2010 Bcl-6 and NF-κB cistromes mediate opposing regulation of the innate immune response. Genes Dev 24: 2760–2765 21106671 10.1101/gad.1998010 PMC3003193

[B3] Bhatt DM, Pandya-Jones A, Tong AJ, Barozzi I, Lissner MM, Natoli G, Black DL, Smale ST 2012 Transcript dynamics of proinflammatory genes revealed by sequence analysis of subcellular RNA fractions. Cell 150: 279–290 22817891 10.1016/j.cell.2012.05.043 PMC3405548

[B4] Boehm MF, Zhang L, Badea BA, White SK, Mais DE, Berger E, Suto CM, Goldman ME, Heyman RA 1994 Synthesis and structure–activity relationships of novel retinoid X receptor-selective retinoids. J Med Chem 37: 2930–2941 8071941 10.1021/jm00044a014

[B5] Boehm MF, Zhang L, Zhi L, McClurg MR, Berger E, Wagoner M, Mais DE, Suto CM, Davies JA, Heyman RA, 1995 Design and synthesis of potent retinoid X receptor selective ligands that induce apoptosis in leukemia cells. J Med Chem 38: 3146–3155 7636877 10.1021/jm00016a018

[B6] Boergesen M, Pedersen TA, Gross B, van Heeringen SJ, Hagenbeek D, Bindesboll C, Caron S, Lalloyer F, Steffensen KR, Nebb HI, 2012 Genome-wide profiling of liver X receptor, retinoid X receptor, and peroxisome proliferator-activated receptor α in mouse liver reveals extensive sharing of binding sites. Mol Cell Biol 32: 852–867 22158963 10.1128/MCB.06175-11 PMC3272984

[B7] Bonn S, Zinzen RP, Girardot C, Gustafson EH, Perez-Gonzalez A, Delhomme N, Ghavi-Helm Y, Wilczynski B, Riddell A, Furlong EE 2012 Tissue-specific analysis of chromatin state identifies temporal signatures of enhancer activity during embryonic development. Nat Genet 44: 148–156 22231485 10.1038/ng.1064

[B8] Brazda P, Krieger J, Daniel B, Jonas D, Szekeres T, Langowski J, Toth K, Nagy L, Vamosi G 2014 Ligand binding shifts highly mobile retinoid x receptor to the chromatin-bound state in a coactivator-dependent manner, as revealed by single-cell imaging. Mol Cell Biol 34: 1234–1245 24449763 10.1128/MCB.01097-13 PMC3993562

[B9] Calkin AC, Tontonoz P 2012 Transcriptional integration of metabolism by the nuclear sterol-activated receptors LXR and FXR. Nat Rev Mol Cell Biol 13: 213–224 22414897 10.1038/nrm3312 PMC3597092

[B10] Carroll JS, Liu XS, Brodsky AS, Li W, Meyer CA, Szary AJ, Eeckhoute J, Shao W, Hestermann EV, Geistlinger TR, 2005 Chromosome-wide mapping of estrogen receptor binding reveals long-range regulation requiring the forkhead protein FoxA1. Cell 122: 33–43 16009131 10.1016/j.cell.2005.05.008

[B11] Chakravarti D, LaMorte VJ, Nelson MC, Nakajima T, Schulman IG, Juguilon H, Montminy M, Evans RM 1996 Role of CBP/P300 in nuclear receptor signalling. Nature 383: 99–103 8779723 10.1038/383099a0

[B12] Core LJ, Waterfall JJ, Lis JT 2008 Nascent RNA sequencing reveals widespread pausing and divergent initiation at human promoters. Science 322: 1845–1848 19056941 10.1126/science.1162228 PMC2833333

[B13] Germain P, Iyer J, Zechel C, Gronemeyer H 2002 Co-regulator recruitment and the mechanism of retinoic acid receptor synergy. Nature 415: 187–192 11805839 10.1038/415187a

[B14] Hah N, Danko CG, Core L, Waterfall JJ, Siepel A, Lis JT, Kraus WL 2011 A rapid, extensive, and transient transcriptional response to estrogen signaling in breast cancer cells. Cell 145: 622–634 21549415 10.1016/j.cell.2011.03.042 PMC3099127

[B15] Hah N, Murakami S, Nagari A, Danko CG, Kraus WL 2013 Enhancer transcripts mark active estrogen receptor binding sites. Genome Res 23: 1210–1223 23636943 10.1101/gr.152306.112 PMC3730096

[B16] Heintzman ND, Stuart RK, Hon G, Fu Y, Ching CW, Hawkins RD, Barrera LO, Van Calcar S, Qu C, Ching KA, 2007 Distinct and predictive chromatin signatures of transcriptional promoters and enhancers in the human genome. Nat Genet 39: 311–318 17277777 10.1038/ng1966

[B17] Jin Q, Yu LR, Wang L, Zhang Z, Kasper LH, Lee JE, Wang C, Brindle PK, Dent SY, Ge K 2011 Distinct roles of GCN5/PCAF-mediated H3K9ac and CBP/p300-mediated H3K18/27ac in nuclear receptor transactivation. EMBO J 30: 249–262 21131905 10.1038/emboj.2010.318 PMC3025463

[B18] Kersten S, Mandard S, Tan NS, Escher P, Metzger D, Chambon P, Gonzalez FJ, Desvergne B, Wahli W 2000 Characterization of the fasting-induced adipose factor FIAF, a novel peroxisome proliferator-activated receptor target gene. J Biol Chem 275: 28488–28493 10862772 10.1074/jbc.M004029200

[B19] Kim TK, Hemberg M, Gray JM, Costa AM, Bear DM, Wu J, Harmin DA, Laptewicz M, Barbara-Haley K, Kuersten S, 2010 Widespread transcription at neuronal activity-regulated enhancers. Nature 465: 182–187 20393465 10.1038/nature09033 PMC3020079

[B20] Koch F, Andrau JC 2011 Initiating RNA polymerase II and TIPs as hallmarks of enhancer activity and tissue-specificity. Transcription 2: 263–268 22223044 10.4161/trns.2.6.18747 PMC3265787

[B21] Koch CM, Andrews RM, Flicek P, Dillon SC, Karaoz U, Clelland GK, Wilcox S, Beare DM, Fowler JC, Couttet P, 2007 The landscape of histone modifications across 1% of the human genome in five human cell lines. Genome Res 17: 691–707 17567990 10.1101/gr.5704207 PMC1891331

[B22] Lam MT, Cho H, Lesch HP, Gosselin D, Heinz S, Tanaka-Oishi Y, Benner C, Kaikkonen MU, Kim AS, Kosaka M, 2013 Rev–Erbs repress macrophage gene expression by inhibiting enhancer-directed transcription. Nature 498: 511–515 23728303 10.1038/nature12209 PMC3839578

[B23] Lefterova MI, Steger DJ, Zhuo D, Qatanani M, Mullican SE, Tuteja G, Manduchi E, Grant GR, Lazar MA 2010 Cell-specific determinants of peroxisome proliferator-activated receptor γ function in adipocytes and macrophages. Mol Cell Biol 30: 2078–2089 20176806 10.1128/MCB.01651-09 PMC2863586

[B24] Mangelsdorf DJ, Borgmeyer U, Heyman RA, Zhou JY, Ong ES, Oro AE, Kakizuka A, Evans RM 1992 Characterization of three RXR genes that mediate the action of 9-cis retinoic acid. Genes Dev 6: 329–344 1312497 10.1101/gad.6.3.329

[B25] Merkenschlager M, Odom DT 2013 CTCF and cohesin: linking gene regulatory elements with their targets. Cell 152: 1285–1297 23498937 10.1016/j.cell.2013.02.029

[B26] Miele A, Gheldof N, Tabuchi TM, Dostie J, Dekker J 2006 Mapping chromatin interactions by chromosome conformation capture. Curr Protoc Mol Biol 74: 21.11.1–21.11.20 10.1002/0471142727.mb2111s74 18265379

[B27] Mortazavi A, Williams BA, McCue K, Schaeffer L, Wold B 2008 Mapping and quantifying mammalian transcriptomes by RNA-seq. Nat Methods 5: 621–628 18516045 10.1038/nmeth.1226 PMC13303166

[B28] Movahedi B, Gysemans C, Jacobs-Tulleneers-Thevissen D, Mathieu C, Pipeleers D 2008 Pancreatic duct cells in human islet cell preparations are a source of angiogenic cytokines interleukin-8 and vascular endothelial growth factor. Diabetes 57: 2128–2136 18492788 10.2337/db07-1705 PMC2494672

[B29] Nagy L, Saydak M, Shipley N, Lu S, Basilion JP, Yan ZH, Syka P, Chandraratna RA, Stein JP, Heyman RA, 1996 Identification and characterization of a versatile retinoid response element (retinoic acid receptor response element-retinoid X receptor response element) in the mouse tissue transglutaminase gene promoter. J Biol Chem 271: 4355–4365 8626785 10.1074/jbc.271.8.4355

[B30] Nagy L, Szanto A, Szatmari I, Szeles L 2012 Nuclear hormone receptors enable macrophages and dendritic cells to sense their lipid environment and shape their immune response. Physiol Rev 92: 739–789 22535896 10.1152/physrev.00004.2011

[B31] Nielsen R, Pedersen TA, Hagenbeek D, Moulos P, Siersbaek R, Megens E, Denissov S, Borgesen M, Francoijs KJ, Mandrup S, 2008 Genome-wide profiling of PPARγ:RXR and RNA polymerase II occupancy reveals temporal activation of distinct metabolic pathways and changes in RXR dimer composition during adipogenesis. Genes Dev 22: 2953–2967 18981474 10.1101/gad.501108 PMC2577787

[B32] Ostuni R, Piccolo V, Barozzi I, Polletti S, Termanini A, Bonifacio S, Curina A, Prosperini E, Ghisletti S, Natoli G 2013 Latent enhancers activated by stimulation in differentiated cells. Cell 152: 157–171 23332752 10.1016/j.cell.2012.12.018

[B33] Pott S, Kamrani NK, Bourque G, Pettersson S, Liu ET 2012 PPARG binding landscapes in macrophages suggest a genome-wide contribution of PU.1 to divergent PPARG binding in human and mouse. PLoS ONE 7: e48102 23118933 10.1371/journal.pone.0048102 PMC3485280

[B34] Repa JJ, Turley SD, Lobaccaro JA, Medina J, Li L, Lustig K, Shan B, Heyman RA, Dietschy JM, Mangelsdorf DJ 2000 Regulation of absorption and ABC1-mediated efflux of cholesterol by RXR heterodimers. Science 289: 1524–1529 10968783 10.1126/science.289.5484.1524

[B35] Schwartz K, Lawn RM, Wade DP 2000 ABC1 gene expression and ApoA-I-mediated cholesterol efflux are regulated by LXR. Biochem Biophys Res Commun 274: 794–802 10924356 10.1006/bbrc.2000.3243

[B36] Sofueva S, Yaffe E, Chan WC, Georgopoulou D, Vietri Rudan M, Mira-Bontenbal H, Pollard SM, Schroth GP, Tanay A, Hadjur S 2013 Cohesin-mediated interactions organize chromosomal domain architecture. EMBO J 32: 3119–3129 24185899 10.1038/emboj.2013.237 PMC4489921

[B37] Stadhouders R, Kolovos P, Brouwer R, Zuin J, van den Heuvel A, Kockx C, Palstra RJ, Wendt KS, Grosveld F, van Ijcken W, 2013 Multiplexed chromosome conformation capture sequencing for rapid genome-scale high-resolution detection of long-range chromatin interactions. Nat Protoc 8: 509–524 23411633 10.1038/nprot.2013.018

[B38] Szanto A, Narkar V, Shen Q, Uray IP, Davies PJ, Nagy L 2004 Retinoid X receptors: X-ploring their (patho)physiological functions. Cell Death Differ 11: S126–S143 15608692 10.1038/sj.cdd.4401533

[B39] Szanto A, Balint BL, Nagy ZS, Barta E, Dezso B, Pap A, Szeles L, Poliska S, Oros M, Evans RM, 2010 STAT6 transcription factor is a facilitator of the nuclear receptor PPARγ-regulated gene expression in macrophages and dendritic cells. Immunity 33: 699–712 21093321 10.1016/j.immuni.2010.11.009 PMC3052437

[B40] Szeles L, Poliska S, Nagy G, Szatmari I, Szanto A, Pap A, Lindstedt M, Santegoets SJ, Ruhl R, Dezso B, 2010 Research resource: transcriptome profiling of genes regulated by RXR and its permissive and nonpermissive partners in differentiating monocyte-derived dendritic cells. Methods Enzymol 24: 2218–2231 10.1210/me.2010-0215 PMC3051201 20861222

[B41] Thurman RE, Rynes E, Humbert R, Vierstra J, Maurano MT, Haugen E, Sheffield NC, Stergachis AB, Wang H, Vernot B, 2012 The accessible chromatin landscape of the human genome. Nature 489: 75–82 22955617 10.1038/nature11232 PMC3721348

[B42] Visel A, Rubin EM, Pennacchio LA 2009 Genomic views of distant-acting enhancers. Nature 461: 199–205 19741700 10.1038/nature08451 PMC2923221

[B43] Wang D, Garcia-Bassets I, Benner C, Li W, Su X, Zhou Y, Qiu J, Liu W, Kaikkonen MU, Ohgi KA, 2011 Reprogramming transcription by distinct classes of enhancers functionally defined by eRNA. Nature 474: 390–394 21572438 10.1038/nature10006 PMC3117022

[B44] Welboren WJ, van Driel MA, Janssen-Megens EM, van Heeringen SJ, Sweep FC, Span PN, Stunnenberg HG 2009 ChIP-seq of ERα and RNA polymerase II defines genes differentially responding to ligands. EMBO J 28: 1418–1428 19339991 10.1038/emboj.2009.88 PMC2688537

[B45] Zentner GE, Scacheri PC 2012 The chromatin fingerprint of gene enhancer elements. J Biol Chem 287: 30888–30896 22952241 10.1074/jbc.R111.296491 PMC3438921

[B46] Zhang XK, Lehmann J, Hoffmann B, Dawson MI, Cameron J, Graupner G, Hermann T, Tran P, Pfahl M 1992 Homodimer formation of retinoid X receptor induced by 9-*cis* retinoic acid. Nature 358: 587–591 1323763 10.1038/358587a0

